# Systemic and Ophthalmic Manifestations in Different Types of Refractive Errors in Patients with Down Syndrome

**DOI:** 10.3390/medicina58080995

**Published:** 2022-07-26

**Authors:** Antonela Ljubic, Vladimir Trajkovski, Branislav Stankovic, Biljana Tojtovska, Andrea Langmann, Galina Dimitrova, Ivana Jovanovic, Milorad Tesic

**Affiliations:** 1 Private Polyclinic “Medika Plus”, 1000 Skopje, North Macedonia; 2 Institute of Special Education and Rehabilitation, Faculty of Philosophy, University “Ss. Cyril and Methodius”, 1000 Skopje, North Macedonia; vladotra@fzf.ukim.edu.mk; 3 Institute of Ophthalmology, University Clinical Center of Serbia, 11000 Belgrade, Serbia; branislav.stankovic@icloud.com; 4 Faculty of Medicine, University of Belgrade, 11000 Belgrade, Serbia; misa.tesic@gmail.com; 5 Faculty of Computer Science and Engineering, University “Ss. Cyril and Methodius”, 1000 Skopje, North Macedonia; biljana.tojtovska@finki.ukim.mk; 6 Department of Ophthalmology, Medical University Graz, 8010 Graz, Austria; andrea.langmann@medunigraz.at; 7 Department of Ophthalmology, General City Hospital, 1000 Skopje, North Macedonia; galinadimi@gmail.com; 8 Clinic for Cardiology, University Clinical Center of Serbia, 11000 Belgrade, Serbia; ivana170679@gmail.com

**Keywords:** Down syndrome, systemic manifestations, nystagmus, iris stromal atrophy, astigmatism

## Abstract

*Background and Objective***:** This study aims to investigate the prevalence of systemic and ophthalmic manifestations in different refractive groups in children and young adults with Down syndrome (DS). *Materials and Methods*: The study was a population-based, cross-sectional study that included 141 Caucasian children and young adults with DS. They were classified into the following three groups: myopia DS group (37 subjects, mean age 15.8 years), emmetropia DS group (41 subjects, mean age 11.7 years) and hyperopia DS group (63 subjects, mean age 10.9 years). The participants underwent inspection, slit-lamp examination, cycloplegic refraction, ocular alignment and ocular motility examination. Ten systemic manifestations were analyzed. *Results*: There was no difference in the prevalence of any systemic manifestations between the groups. Considering the ophthalmic manifestations, there was statistical difference in the distribution of proportions among the three groups for nystagmus (*p* = 0.011), iris-stromal atrophy (*p* = 0.048) and strabismus (*p* = 0.031). The prevalence of strabismus in our DS myopia group was 35.1%, and in DS hyperopia group 38.1%. *Conclusions*: The results of our study suggest that DS children and young adults with any refractive error do not have a higher chance of additional systemic manifestations. Myopia in DS was associated with a higher prevalence of nystagmus and iris stromal atrophy, whereas astigmatism was found to be more frequent in hyperopia.

## 1. Introduction

Down syndrome (DS) is a genetic disorder well known for a high prevalence of refractive errors. The exact reason for this frequent occurrence of refractive errors in DS is still unknown. In the current literature, many studies on DS patients have reported a high prevalence of refractive errors, ranging between 30% and 98% [1,2,3,4,5]. In early infancy, the mean refractive error does not differ between the general population and DS population. In DS, a variety of refractive errors arise with increasing age (possibly due to failed emmetropization) [2,3].

The most common refractive error in DS is hyperopia. The prevalence of hyperopia in DS ranges from 26% to 69.1% [5,6]. The prevalence of hyperopia in the Slovenian Caucasian DS cohort is 36.9% [7], and in the Croatian-Dalmatian Caucasian DS cohort 35% [8]. Our previously published results on the Macedonian-Croatian Caucasian DS cohort showed a 55.2% prevalence of hyperopia [9], similar to the Italian (59%) [10] and Japanese DS cohort (58.5%) [11].

The prevalence of myopia in the DS population in different studies ranges from 6.3% to 40.1% [12,13]. The largest DS study ever published on an American DS cohort showed a prevalence of myopia of 22.5% [4]. The prevalence of DS myopia in our region for the Slovenian Caucasian DS cohort was 24.6% [7], and in the Macedonian-Croatian Caucasian cohort, it was 20.7% [9].

Presence of systemic diseases (such as congenital heart defects (CHD), hypotony, hypothyroidism, hearing loss and others) in patients with Down syndrome and the associated ophthalmic pathologies have rarely been analyzed in the current literature. Stirn-Kranjc et al. [7], Bromham et al. [14] and Affifi et al. [15] all found that CHD in DS were associated with myopia and nystagmus. Thus, the aim of this study was to examine the prevalence of systemic diseases and ocular manifestations in different refractive groups of children and young adults with DS.

## 2. Methods

This was a population-based, cross-sectional study. A total of 184 Caucasian children and young adults with DS were examined. Patients were recruited from the special education system, social services, as well as from parental associations in the region of North Macedonia and Croatia. Based on the exclusion criteria (congenital cataract, dense cataract, aphakia, pseudophakia and advanced keratoconus), only 141 children and young adults (1.9–33.9 years) were included in our study. Patients were divided into the following three groups: myopia DS group (37 patients, mean age 15.8 years), emmetropia DS group (41 patients, mean age 11.7 years) and hyperopia DS group (63 patients, mean age 10.9 years).

In the period from March 2007 until July 2009, the patients were evaluated at the private polyclinics “Medika plus” in Skopje, North Macedonia and “Svjetlost” in Zagreb, Croatia. Eye exams were also carried out at several other local private clinics located in eight towns of North Macedonia and in three towns in Croatia. When patients were recruited, North Macedonia did not have a national registry of people with Down syndrome, so the Institute of Special Education and Rehabilitation officially contacted social services in 11 major towns. The services of the capital and 8 towns provided the contacts of all Down syndrome individuals in their area. The services in 2 other cities declined to cooperate. The data-obtaining process in Croatia was similar. Croatia, at the time, had a strong network of Down parental associations. The Down patients were recruited through these associations, from the capital and 3 major regions. An effort was made to obtain data from additional regions, but it was not successful. Other regions in both countries were not included in this phase of the research for various reasons (time constraints, funding but also lack of contacts and expected difficulties to organize medical examinations).

North Macedonia (country in the south-east of Europe) and Croatia (country in Adriatic region in Europe) are two separate ethnic countries, with Caucasian populations. In the present Caucasian Down cohort, there were only ethnic and religious differences. All parents of Macedonian children were of Macedonian origin, except for the two mothers who were from Bulgaria. All Croatian children were of Croatian origin.

Diagnosis of DS was based either on patient clinical characteristics or cytogenetic analyses.

All patients were examined for ocular findings by one of the authors (A.Lj.). Ocular examination included inspection, slit-lamp examination, assessment of cycloplegic refraction, ocular alignment, and ocular motility.

### 2.1. Systemic Manifestations

Protocols for general examination [16,17] were prepared. In each patient, the following 10 systemic manifestations were evaluated: presence of CHD, ear-nose-throat (ENT) disorders, oro-dental findings, thyroid function, diabetes mellitus, dermatological disease, gastrointestinal (GI) disorders, orthopedic disorders, neuro-psychiatric disorders and overweight (obesity).

Cardiac and thyroid status were assessed accordingly. For each patient, a comprehensive cardiac ultrasound was performed as well as laboratory testing for thyroid function (free triiodothyronine T3, free levorotatory thyroxine T4 and thyrotropin). Classification of different types of CHD was based on echocardiographic findings, including (1) ventricular septal defect; (2) atrial septal defect; (3) atrioventricular septal defect; (4) ductus arteriosus; (5) mitral valve prolapse and (6) tetralogy of Fallot. Examination for ENT disorders was conducted with audiometry in patients over 4 years of age. Body mass index (BMI) was used for diagnosis of overweight and obesity. BMI was calculated as weight/height and expressed as kg/m^2^.

### 2.2. External Eye, Anterior and Medial Ocular Segment

Presence of epicanthic folds, epiblepharon and hypertelorismus was established by inspection of the external eye. Occurrence of any ocular manifestation of the palpebrae, conjunctiva or cornea, as well as iris and lens, was assessed by biomicroscopy.

### 2.3. Objective Refraction

Cycloplegic refraction was performed after administration of cyclopentolate 1% solution (one drop of the solution repeated three to five times). In this study, the method of photorefraction (Potec Auto-Ref-Keratometer PRK-5000, Daejeon, Korea) was used.

For each patient, spherical equivalent and power and axis of cylinder were recorded. The spherical equivalent was calculated by adding the sum of the sphere power with half of the cylinder power. Emmetropia was defined as the refractive error between −0.75 diopter (D) and +0.75 D spherical equivalent. Myopia was defined as less than −0.75 D spherical equivalent, and hyperopia was defined as more than +0.75 D spherical equivalent. Low grade hyperopia was defined as +1.00 D to +2.75 D spherical equivalent, moderate hyperopia as +3.00 D to +5.75 D spherical equivalent and high-grade hyperopia as ≥+6.00 D spherical equivalent. Respectively, myopia was also categorized as low-grade, moderate and high-grade. Clinically significant astigmatism was defined as a refractive error ≥ 1.00 D of the cylinder. In the evaluation of the astigmatism group, minus form of the cylinder was used. The axis of astigmatism was classified as follows: WTR (“with the rule”), ATR (“against the rule”) and OBL (oblique astigmatism) or axis between 100–170 and 10–80. Eyes with a cylindrical power of <1.00 D were excluded from the astigmatism group.

### 2.4. Eye Alignment

Eye alignment was assessed using Hirschberg’s test (corneal light reflex test) and cover test. A cover test was also performed using an accommodative fixation target. Both distance and near ocular alignment were tested with optical correction, if prescribed. Classification of deviations, including infantile esodeviations, acquired esodeviations, exodeviations and vertical deviations, were carried out according to the Royal College of Ophthalmologists (RCOPHTH) guidelines [18]. Infantile esodeviations were defined as congenital esodeviations with the onset before 6 months of age reported by the parent. All other cases of esodeviations were classified as acquired esodeviations. Intermittent exotropia and manifest exotropia were defined as exodeviation. The presence of nystagmus was noted (latent or manifest).

### 2.5. Statistical Analysis

All collected data were categorized as categorical and numerical data. Descriptive statistics are presented with frequency tables and graphics, and mean values, percentages, corresponding standard deviation (SD) and standard error (SE) were reported. The test for the distribution of proportions was applied to the corresponding 2 × 2 and 2 × 3 contingency tables (the exact test was applied where appropriate). The Kruskal–Wallis test was used to test for difference in means in the three groups. The alpha level of statistical significance was set at 0.05 and reported *p* values were not calculated with a correction (please refer to the section Study Limitations for more details). Study results were processed in the statistical software package R (version 4.0.2; R Foundation for Statistical Computing, Vienna, Austria).

## 3. Results

Demographic data on DS patients with myopia, emmetropia and hyperopia are presented in Table 1. Cytogenetic confirmation of DS diagnosis in the myopia group was obtained in 32.4% (*n* = 12), in the emmetropia group in 56.1% (*n* = 23) and in the hyperopia group in 46% (*n* = 29) of patients.

Different systemic manifestations across different refractive groups (myopia, emmetropia and hyperopia) are presented in Table 2. There was no difference in prevalence of any systemic manifestations between the groups. Some of the systemic manifestations were very uncommon, while oro-dental systemic manifestations were the most frequent.

**Figure 1:**
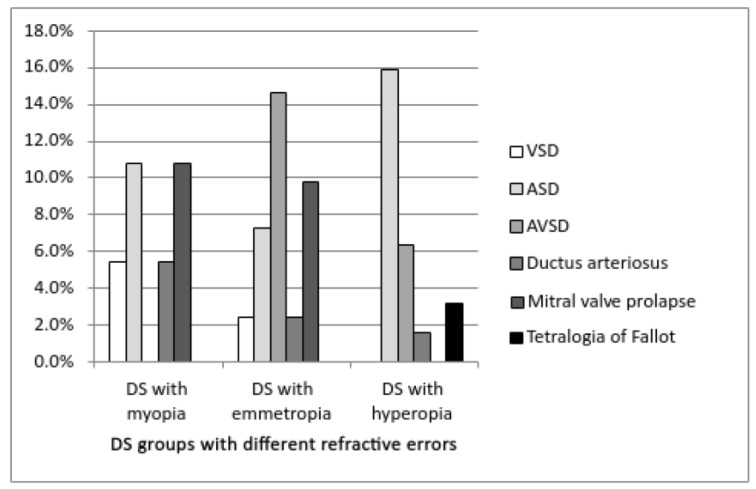
Distribution of different types of congenital heart defects (CHD) in myopia, emmetropia and hyperopia DS group (VSD—ventricular septal defect, ASD—atrial septal defect, AVSD—atrioventricular septal defect).

The distribution of different types of CHD across DS groups (Figure 1) showed that the atrial septal defect (ASD) and mitral valve prolapse were the most common in the myopia group, atrioventricular septal defect (AVSD) in the emmetropia group, while ASD was the most frequent in the hyperopia DS group.

Ophthalmic manifestations in the DS refractive groups are presented in Table 3, including the prevalence of nystagmus, iris stromal atrophy and strabismus. The results of the additional test of distribution of these three ophthalmic entities in any of the two DS groups are shown in Table 4.

Range power (D) (spherical equivalent) and age range (years) in the myopia and hyperopia DS group are presented in Table 5 and Table 6.

The mean spherical equivalent in the myopia DS group was −7.92 D (SD 4.79 D), in the emmetropia DS group +0.16 D (SD 0.47 D) and in the hyperopia DS group +4.00 D (SD 2.12 D).

The mean numeric value of astigmatism in the DS group with myopia was −2.23 D (SD 1.02 D) of the cylinder (25 out of 37 patients), and in the DS hyperopia group −1.72 D (SD 0.75 D) of the cylinder (52 out of 63 patents). The most common type of astigmatism in all three refractive groups was oblique astigmatism.

The distribution of the different types of strabismus as a function of the different ranges of refractive error is presented in Table 7. The most common type of strabismus was acquired esotropia (60.4%).

## 4. Discussion

There was no difference in prevalence of the various systemic manifestations across the three DS refractive groups (myopia, emmetropia, hyperopia). In addition, no statistical difference was found in the distribution of CHD between the three refractive DS groups, which is in agreement with our previous results [19]. Prior studies on smaller Caucasian DS cohorts [5,7,14] found a correlation between CHD and myopia, nystagmus and esotropia. Afifi et al. [15] found a correlation between CHD and myopia in an Egypt DS cohort, with no Brushfield spots present. In our former study [19], we found a correlation between CHD and Brushfield spots.

By analyzing the prevalence of different ophthalmic manifestations across the three DS refractive groups, we found that nystagmus and iris stromal atrophy were the most common in the myopia group. According to the current literature, prevalence of nystagmus in DS individuals ranges from 3.3% to 33.3% [15,20]. As previously noted, in the present study, nystagmus was the most common in the myopia group (18.9% vs. 1.6% in the hyperopia group). The exact cause of nystagmus in the DS population is still unknown [15]. PAX6 locus ophthalmic sequel of mutations located on chromosome 11p13 may be associated with extreme refractive errors, especially high myopia, nystagmus and iris hypoplasia, although the underlying mechanism is not entirely clear [21]. Correlation between myopia and nystagmus was found in the Slovenian DS cohort [7]. Wagner AR et al. [22] also reported an association between nystagmus and myopia. Nystagmus represents one of the ocular features in DS, possibly caused by the yet undetected central nervous system anomaly or by unrecognized sensory retinal abnormalities.

Iris anomalies in DS include anterior stromal hypoplasia and Brushfield spots, which are unchanged with age [23]. Previous publications have reported that iris anomalies are more common in individuals with light-colored irises [5,21]. It is plausible that abnormal extracellular matrix production and vascular abnormalities lead to tissue hypoxia, causing degenerative iris tissue changes (iris stromal atrophy) [24,25].

Iris stromal hypoplasia was first reported by Lowe et al. [26], speculating that it might be even more characteristic for DS than Brushfield spots. Dark brown irises fail to show this peripheral thinning, while it tends to be diffuse in blue eyes and patchy in hazel and light brown eyes. The prevalence of iris stromal hypoplasia in the DS population is reported to range between 22% and 94%, while in the non-DS population, it is estimated at 9% [23]. This variability in prevalence is probably due to the variable proportion of dark-eyed individuals within the studied DS population. In our study, iris stromal atrophy was most common in the myopia group (48.6%). The reason for the increased prevalence of iris stromal thinning remains uncertain. Iris thinning may be considered to be a part of an early aging process that seems to affect some individuals with DS. It was previously reported that those who were homozygous for the D2 allele of locus ISA on chromosome 4 developed the iris stromal atrophy phenotype [27]. In addition, the EDICT syndrome gene located in the region of chromosome 15 between markers D15S993 and D15S202 may increase insight into a broad range of disorders affecting the iris, lens, corneal stroma and endothelium [28].

The present study showed that the prevalence of astigmatism was higher in the hyperopia group (82.5%). In DS infants, the prevalence of astigmatism ranges between 26% and 53% [1,2,7], and increases with age up to 72.4% [9] in DS children and young adults. It was previously reported that oblique astigmatism was the most common type of astigmatism in all refractive groups among young adults with DS [28]. The thinning of corneal stroma may explain the steeper cornea and high frequency of astigmatism in DS as a result of lower corneal rigidity [29].

The prevalence of strabismus in our DS myopia group was 35.1%, while in the DS hyperopia group, it was 38.1%. Acquired esotropia was the most common type of strabismus and it was most frequent in high-grade myopia and in moderate hyperopia with an equal distribution among the two entities. Haugen et al. [30] and Cregg et al. [3] have also reported strabismus in all refractive groups among their cohort of children with DS. Hyperopia is the most common refractive error in DS individuals, with the degree of myopia that can be extremely high [5,25,31]. Most of the clinical characteristics of nonaccommodative esotropia in myopia are similar to those associated with emmetropia or hyperopia.

## 5. Study Limitations

A limitation of our study was the lack of results for posterior ocular segment examination (because a significant number of patients did not cooperate sufficiently for reliable evaluation). Even though there are multiple comparisons, no adjustments were made to the *p*-values, i.e., all *p* values in this paper are reported without corrections. Some previous studies reported only the significant results and not the number of the conducted tests, while some studies did not report any adjustments. For easier comparison of all these results, we decided to report the *p* values of all tests without adjustments.

## 6. Conclusions

The present study demonstrated that DS children and young adults with any refractive errors did not have a higher risk of additional systemic manifestations in comparison to DS individuals with emmetropia. Myopia was associated with a higher prevalence of nystagmus and iris-stromal atrophy. Our cohort of DS children and young adults showed equal frequency of strabismus in the high-grade myopia and moderate hyperopia (over +3.00 D) groups. Hyperopia in our DS cohort was associated with a higher prevalence of astigmatism.

## Figures and Tables

**Table 1:** Demographic characteristics of children and young adults with Down syndrome and ocular manifestations (myopia, emmetropia and hyperopia).

Characteristic	DS with Myopia (*n* = 37)	DS with Emmetropia (*n* = 41)	DS with Hyperopia (*n* = 63)	*p* Value
Age, mean years (SD)	15.8 (8.0)	11.7 (7.7)	10.9 (6.0)	0.009 ^(a)^
Maternal age, mean years (SD)	28.5 (5.5)	28.2 (6.8)	29.6 (6.1)	0.349 ^(a)^
Paternal age, mean years (SD)	31.8 (6.0)	31.7 (7.0)	33.2 (6.8)	0.442 ^(a)^
Male sex % (SE)	56.8 (8.1)	43.9 (7.8)	65.1 (6)	0.103 ^(b)^

^(a)^—Test for difference in means in the three groups (Kruskal–Wallis). ^(b)^—Test for difference in distribution of proportions in the three groups (with Fisher exact test where appropriate). DS—Down syndrome; SD—standard deviation; SE—standard error.

**Table 2:** Systemic manifestations in children and young adults with Down syndrome and ocular manifestations (myopia, emmetropia and hyperopia).

Systemic Manifestations	DS with Myopia (*n* = 37) % (SE)	DS with Emmetropia (*n* = 41) % (SE)	DS with Hyperopia (*n* = 63) % (SE)	*p* Value
Congenital heart defects	32.4 (7.7)	36.6 (7.5)	27 (5.6)	0.577 ^(b)^
Ear-nose-throat disorders	18.9 (6.4)	12.2 (5.1)	9.5 (3.7)	0.400 ^(b)^
Oro-dental disease	54.1 (8.2)	51.2 (7.8)	47.6 (6.3)	0.817 ^(b)^
Thyroid dysfunction	10.8 (5.1)	19.5 (6.2)	12.7 (4.2)	0.493 ^(b)^
Diabetes mellitus	0 (0)	2.4 (2.4)	0 (0)	0.553 ^(b)^
Dermatological disease	24.3 (7.1)	12.2 (5.1)	33.3 (5.9)	0.051 ^(b)^
Gastrointestinal disorders	13.5 (5.6)	7.3 (4.1)	6.3 (3.1)	0.463 ^(b)^
Musculosceletal (orthopedic) disorders	16.2 (6.1)	22.0 (6.5)	17.5 (4.8)	0.779 ^(b)^
Neuropsychiatric disorders	16.2 (6.1)	9.8 (4.6)	4.8 (2.7)	0.152 ^(b)^
Overweight	18.9 (6.4)	17.1 (5.9)	12.7 (4.2)	0.677 ^(b)^

^(b)^—Test for difference in distribution of proportions in the three groups (with Fisher exact test where appropriate). DS—Down syndrome; SE—standard error.

**Table 3:** Ophthalmic manifestations in children and young adults with Down syndrome.

	DS with Myopia (*n* = 37) % (SE)	DS with Emmetropia (*n* = 41) % (SE)	DS with Hyperopia	*p*-Value
			(*n* = 63) % (SE)	
Ocular alignment				
Strabismus	35.1 (7.8)	14.6 (5.5)	38.1 (6.1)	0.031 ^(b)^
Nystagmus	18.9 (6.4)	12.2 (5.1)	1.6 (1.6)	0.011 ^(b)^
Astigmatism	67.6 (7.7)	0 (0)	82.5 (4.8)	0.086 ^(c)^
Astigmatism (type)				
Oblique	43.2 (8.1)	0 (0)	41.3 (6.2)	
ATR	2.7 (2.7)	0 (0)	14.3 (4.4)	
WTR	21.6 (6.8)	0 (0)	27.0 (5.6)	
No astigmatism	32.4 (7.7)	100 (0)	17.5 (4.8)	
Anterior and medial segment				
Epiblepharon	32.4 (7.7)	31.7 (7.3)	25.4 (5.5)	0.686 ^(b)^
Epicanthus	27.0 (7.3)	26.8 (6.9)	25.4 (5.5)	0.979 ^(b)^
Hypertelorism	5.4 (3.7)	9.8 (4.6)	4.8 (2.7)	0.617 ^(b)^
Conjunctivitis	8.1 (4.5)	2.4 (2.4)	14.3 (4.4)	0.117 ^(b)^
Blepharitis	13.5 (5.6)	22.0 (6.5)	19 (4.9)	0.622 ^(b)^
Blepharocunjuctivitis	8.1 (4.5)	2.4 (2.4)	1.6 (1.6)	0.171 ^(b)^
Cornea changes	0 (0)	2.4 (2.4)	0 (0)	-
Glaucoma	0 (0)	0 (0)	0 (0)	-
Brushfield spots	24.3 (7.1)	31.7 (7.3)	22.2 (5.2)	0.545 ^(b)^
Iris stromal atrophy	48.6 (8.2)	24.4 (6.7)	28.6 (5.7)	0.048 ^(b)^
Lens opacitates	13.5 (5.6)	2.4 (2.4)	6.3 (3.1)	0.168 ^(b)^
Iris color				
Brown	56.8 (18.8)	58.5 (18.6)	58.7 (15)	-
Blue	32.4 (11.4)	24.4 (8.9)	30.2 (8.3)	
Green	10.8 (5.7)	17.1 (7.1)	11.1 (4.5)	

^(b)^—Test for difference in distribution of proportions in the three groups (with Fisher exact test where appropriate). ^(c)^—Test for difference in distribution of proportions in two groups (with Fisher exact test where appropriate). DS—Down syndrome; SE—standard error.

**Table 4:** Additional tests for distribution of strabismus, nystagmus and iris stromal atrophy in any of the two groups.

	Myopia vs. Emmetropia	Myopia vs. Hyperopia	Emmetropia vs. Hyperopia
Strabismus	0.035 ^(c)^	0.767 ^(c)^	0.010 ^(c)^
Nystagmus	0.411 ^(c)^	0.004 ^(c)^	0.034 ^(c)^
Iris stromal atrophy	0.026 ^(c)^	0.043 ^(c)^	0.639 ^(c)^

^(c)^—Test for difference in distribution of proportions in two groups (with Fisher exact test where appropriate).

**Table 5:** Age and spherical equivalent distribution in myopia group.

Age Range (Years)	Myopia, Range Power, D			
	Low (−1.00 to −2.75)	Medium (−3.00 to −5.75)	High (≤−6.00)	Total *n* (%)
0–4	1	0	1	2 (5.4)
5–9	1	3	6	10 (27)
10–14	1	1	3	5 (13.5)
≥15	2	5	13	20 (54.1)
Total *n* (%)	5 (13.5)	9 (24.3)	23 (62.2)	37 (100)

**Table 6:** Age and spherical equivalent distribution in hyperopia group.

Age Range (Years)	Hyperopia, Range Power, D			
	Low (+1.00 to +2.75)	Medium (+3.00 to +5.75)	High (≥+6.00)	Total *n* (%)
0–4	7	1	2	10 (15.8)
5–9	8	9	2	19 (30.2)
10–14	5	8	5	18 (28.6)
≥15	5	10	1	16 (25.4)
Total *n* (%)	25 (39.8)	28 (44.4)	10 (15.8)	63 (100)

**Table 7:** Distribution of different types of strabismus as a function of the different ranges of refractive error.

Type of Strabismus	Range Power, D							
	Myopia (*n* = 37)			Emmetropia (*n* = 41) (−0.75 to 0.75)	Hyperopia (*n* = 63)			Total *n* (%)
	Low (−1.00 to −2.75)	Moderate (−3.00 to −5.75)	High (≤−6.00)		Low (+1.00 to +2.75)	Moderate (+3.00 to +5.75)	High (≥+6.00)	
Infantile esotropia	0	0	0	0	2	1	0	3 (7)
Acquired esotropia	0	4	6	4	3	6	3	26 (60.4)
Exodeviations	1	0	1	1	1	5	2	11 (25.6)
Vertical deviations	0	0	1	1	0	1	0	3 (7)
Total *n* (%)	1 (2.3)	4 (9.3)	8 (18.6)	6 (14)	6 (14)	13 (30.2)	5 (11.6)	43

## Data Availability

The data presented in this study are available upon request from the corresponding author. The data are not publicly available due to privacy.
